# Enzymatic Preparation of Gentiooligosaccharides by a Thermophilic and Thermostable β-Glucosidase at a High Substrate Concentration

**DOI:** 10.3390/foods11030357

**Published:** 2022-01-26

**Authors:** Wei Xia, Lingling Sheng, Wanmeng Mu, Yuping Shi, Jing Wu

**Affiliations:** 1 State Key Laboratory of Food Science and Technology, Jiangnan University, 1800 Lihu Avenue, Wuxi 214122, China; weixia@jiangnan.edu.cn (W.X.); sheng_lingling@sina.com (L.S.); wmmu@jiangnan.edu.cn (W.M.); 2 Key Laboratory of Industrial Biotechnology, School of Biotechnology, Ministry of Education, Jiangnan University, 1800 Lihu Avenue, Wuxi 214122, China; 3 International Joint Laboratory on Food Safety, Jiangnan University, 1800 Lihu Avenue, Wuxi 214122, China; 4 Buhler (Changzhou) Machinery Co., Ltd., No. 88 Yunmei Road, Tianmu Lake District, Changzhou 213332, China; syp_jsly@163.com

**Keywords:** gentiooligosaccharides, β-glucosidase, transglycosylation, thermophilic, gentiobiose

## Abstract

Gentiooligosaccharides (GnOS) are a kind of oligosaccharide formed by glucose with β-1-6 glycosidic bonds, which has become a new type of functional oligosaccharide for its unique refreshing bitter taste and valuable probiotic effects. However, the research on the enzymatic preparation of GnOS is not thorough enough. In this study, a GH1 thermophilic β-glucosidase from *Thermotoga* sp. KOL6 was used as a biocatalyst for the synthesis of GnOS. TsBgl1 exhibited excellent thermophilic and thermostable properties by possessing a melting temperature of 101.5 °C and reacting at 80–90 °C efficiently. Its half-life at 90 °C was approximately 5 h, suggesting its high heat resistance as well. TsBgl1 also showed excellent glucose tolerance with an inhibition constant (*K*_i_) of 1720 mM and was stimulated in the presence of 0–900 mM glucose. TsBgl1 showed the highest hydrolytic activity on laminaribiose (Glc-β-1,3-Glc), but mainly synthetized gentiobiose (Glc-β-1,6-Glc) during transglycosylation. By optimizing the reaction conditions and substrate concentration, the highest yield of GnOS synthesized by TsBgl1 reached 144.3 g·L^−1^ when 1000 g·L^−1^ glucose was used as a substrate, which was higher than the highest yield ever reported. The thermophilic and thermostable properties of TsBgl1 were considered to be significant advantages in the industrial production of GnOS, where long periods of high-temperature reactions are required. This study was expected to provide an excellent candidate enzyme for industrial production of GnOS and also provide a reference for studying the transglycosylation of GH1 β-glucosidases.

## 1. Introduction

Gentiooligosaccharides (GnOS) are a kind of oligosaccharide formed by glucose connected by β-1-6 glycosidic bonds. A small amount of natural GnOS exists in plant rhizomes and honey. Its main components are gentiobiose and a small amount of trisaccharide and tetrasaccharide. GnOS is a representative functional oligosaccharide with a unique refreshing bitter taste. Compared with natural bitter substances such as naringin, its bitter taste is softer and more similar to the flavor of chocolate, cocoa, coffee and beer, and can reduce the astringency of fruits and vegetables. This bitter taste makes GnOS a flavor enhancer for some beverages [1], which can increase the richness of food taste by a small amount of addition [2,3]. Compared with functional oligosaccharides such as isomaltooligosaccharides, fructo-oligosaccharide and galactooligosaccharide, GnOS has the characteristics of low viscosity and high tolerance to pH and heat, so it can prevent starch aging and can prolong the shelf life of some other oligosaccharide-intolerant foods. GnOS is also a kind of hygroscopic sugar, which has good water retention to keep some foods properly hydrated and prevent them from being contaminated by microorganisms. It was reported that oligosaccharides containing a (1→6)-β-Glc group, including gentiobiose were resistant to digestion by both the common oral bacterium *Streptococcus mutans* and mammalian intestinal enzymes [4], making GnOS known as a low-digestible sugar [5]. GnOS contains β-1-6 glucosidic bonds that are not easy to be digested and decomposed by the human body, which makes it exhibit excellent intestinal prebiotic effects by entering the human colon and being selectively used by intestinal microorganisms. GnOS with DP 2–3 human showed obvious prebiotic effect with a prebiotic index (PI) of 4.89 on human faecal bacteria under anaerobic conditions at 37 °C [6]. It was also reported that GnOS had an intestinal regulatory function similar to galactooligosaccharides and showed a strong proliferation-promoting effect on *Bifidobacterium* and *Lactobaciiius* species. In addition, the C-terminal alkyl derivatives of GnOS were reported to be used as antineoplastic drugs [7]. At present, as a new type of functional oligosaccharide, GnOS has been widely used in favorite foods, such as chocolate, ice cream, jam, coffee and so on.

Early preparation methods of GnOS were mainly extraction from the stems and roots of gentian, amygdalin hydrolysis or purification from the acid hydrolysis by-products of starch. However, these methods were limited by many conditions, such as high cost and low yield. As a result, the market price of GnOS was on the high side, making it difficult to apply on a large scale [8,9]. In contrast, enzymatic preparation has the advantages of mild reaction conditions, safety, low pollution, low cost and easy separation, which has been the main development trend in the production of GnOS.

The enzymatic preparation of GnOS is mainly achieved by β-glucosidase catalyzed transglycosylation. In the early years, Japanese researchers published the enzymatic production process of converting high concentration glucose solution into GnOS using *Aspergillus niger* β-glucosidase, and realized the large-scale industrial production of GnOS for the first time [10]. The mixture containing GnOS was produced by the transglycoside action of β-glucosidase, and then different GnOS were prepared by different purification methods. So far, only Japanese Food and Chemical Co., Ltd. could produce commercial products of GnOS (Gentose^#^45 and Gentose^#^80), and the output is only 350 t, which led to a large market gap. With the expansion of market demand, the enzymatic preparation of GnOS has attracted increasing attention from researchers, but there is no ideal case report at present.

β-glucosidases (EC3.2.1.21) are a kind of glycoside hydrolase (GH) that can specifically hydrolyze oligosaccharides or glycoside derivatives with β-D-glucoside bond at the non-reducing end of the substrate to release glucose and corresponding aglycons. Most β-glucosidases belong to GH1 and GH3 families, and a small number of β-glucosidases are distributed in GH5, GH9, GH30 and GH116 families [11,12,13,14]. Except for the GH9 family, most of the characterized β-glucosidases adopt a retaining double substitution mechanism [15,16], which includes two catalytic processes [17]. The first step is the glycosylation of the enzyme, that is, the glycosyl-enzyme intermediate is formed by the covalent bonding of the nucleophile residue and the −1 glucosyl unit dissociated from the glycoside substrate. The second step is deglycosylation. Under the action of the acid-base catalytic center of the enzyme, the glucosyl of the glycosyl-enzyme intermediate is transferred to the nucleophilic hydroxyl of the glycosyl receptor, thus completing the double substitution reaction.

It is generally believed that retaining glycoside hydrolases have the potential activities to transfer the glycosyl to the receptor ligands of other non-aqueous molecules in the deglycosylation process of the glycosyl-enzyme covalent complex, thus catalyzing the transglycosylation reaction. β-glucosidase can mediate the transglycosylation and condensation of glucose derivatives, showing synthetic activities of oligosaccharides, alkyl glycosides [13] and vitamin derivatives [18]. The transglycosylation activity and hydrolysis activity of β-glucosidase have been highly valued in industrial technology [19]. LeGao et al. found a β-glucosidase from *Penicillium*, which not only showed the hydrolytic activity of *p*NP-β-Glc and cellobiose, but also could produce sophorose by transglycosylation with glucose as the substrate [20]. In comparison with glycosyltransferases which require expensive nucleotides as substrates, β-glucosidases could synthetize oligosaccharides using cheap substrates such as monosaccharides or disaccharides [21]. Therefore, β-glucosidases have great advantages in industrial synthesis processes.

In the process of GnOS synthesis by β-glucosidases, the most important factor affecting the yield is the transglycosylation activity of the enzyme. β-glucosidase with high transglycosylation activity can effectively increase the synthesis rate of the product and increase the yield of GnOS [10]. At present, most of the β-glucosidases used in the preparation of GnOS belong to the GH3 family, while the studies on the GH1 family are relatively few. However, in recent years, it has been reported that the GH1 β-glucosidases had higher transglycosylation activity than that from the GH3 family [22]. In addition, increasing the substrate concentration of the reaction system can reduce the water activity of the reaction system, thus inhibiting the occurrence of the hydrolysis reaction and promoting the accumulation of transglycosylation products. For large-scale industrial production, the higher the reaction temperature is, the higher the solubility of glucose is, and the lower the viscosity of the reaction solution is, which is more conducive to the transglycosylation reaction [23]. Therefore, β-glucosidases with high-temperature resistance can better adapt to the reaction system with higher substrate concentration in industrial continuous production [24]. Compared with GH3 β-glucosidase with three separated domains, GH1 β-glucosidases are single-domain proteins with smaller and stabler structures, which is also more conducive to industrial production and practical applications.

Based on the above considerations, thermostable GH1 β-glucosidases from thermophiles are good candidate enzymes for the preparation of GnOS with greater application prospects. In this study, GH1 thermophilic β-glucosidase from *Thermotoga* sp. KOL6, TsBgl1, was used as a biocatalyst for the preparation of GnOS. The optimum conditions for the preparation of GnOS and the product composition were investigated, which was expected to provide a new excellent candidate enzyme for the industrial production of GnOS.

## 2. Materials and Methods

### 2.1. Strains and Reagents

Strain *Escherichia coli* JM109, T4DNA ligase, DNA polymerase PrimeSTAR ^®^HS, In-Fusion HD Cloning Plus kit and restriction enzymes were purchased from Dalian BaoBio Co., Ltd. (Dalian, China). The expression host *Bacillus subtilis* WS11 and the expression vector pBSMμL3 of *B. subtilis* were constructed in the early works of our laboratory. Agarose gel DNA recovery kit, PCR purification kit and plasmid extraction kit were purchased from Beijing Tiangen Biochemical Technology Co., Ltd. (Beijing, China). The coding gene of β-glucosidase TsBgl1 and PCR primers were synthesized by Wuxi Tianlin Biotechnology Co., Ltd. (Wuxi, China). Ampicillin (Amp) and kanamycin (Kan) were purchased from Shanghai Jerry Co., Ltd. (Shanghai, China). The substrates 4-nitrophenyl-β-D-glucopyranoside (*p*NP-β-Glc), cellobiose, sophorose, gentiobiose and laminaribiose were purchased from Sigma-Aldrich (St. Louis, MO, USA). GOD glucose detection kit was purchased from Shanghai Rongsheng Biotechnology Co., Ltd. (Shanghai, China). Other conventional reagents are purchased from Sinopharmaceutical Group Chemical Reagent Co., Ltd. (Shanghai, China).

### 2.2. Expression and Purification of Recombinant TsBgl1

According to the codon preference of *B. subtilis*, the coding gene of β-glucosidase TsBgl1 from *Thermotoga* sp. KOL6 (accession number WP_101510358.1) was chemically synthesized after codon optimization. The target gene *TsBgl1* was ligated with the expression vector pBSMμL3 by In-Fusion cloning kit (primers shown in Table S1), and then amplified by the cloning host *E. coli* JM109. The recombinant expression plasmid pBSMμL3-*TsBgl1* was then electro-transformed into the expression host *B. subtilis* WS11 to obtain the recombinant expression strain *B. subtilis*/pBSMμL3-*TsBgl1*. After being shaken in a TB medium for 24 h, the cell of the expression strain was collected by centrifugation, sucked and re-dissolved with pH 6.0 phosphate-citric acid buffer, and then crushed by ultrasonic homogenization (SCIENTZ IID, Scientz, Ningbo, China). Then, the supernatant was centrifuged to obtain a crude enzyme solution of β-glucosidase TsBgl1. The crude enzyme was purified by ammonium sulfate fractional precipitation and Ni^2+^ affinity chromatography. The detailed method was as follows: the crude enzyme solution was sampled on the Ni-NTA column, washed with washing buffer A (50 mM phosphate buffer, 50 mM imidazole, pH 7.4), and eluted by linear gradient elution with buffer B (50 mM phosphate buffer, 500 mM imidazole, pH 7.4). The eluate from the sample tube with the highest purity was collected and dialyzed overnight with the phosphate buffer of pH 6.0, and finally, the purified enzyme solution of TsBgl1 was obtained. The purified TsBgl1 was analyzed by SDS-PAGE gel electrophoresis.

### 2.3. Enzyme Activity Assay

The enzyme activity assays were performed in a 1.0 mL reaction system containing 2 mM *p*NP-β-Glc. The reaction tube was preheated for 5–10 min by water bath in an Eppendorf thermomixer thermostatic homogenizer. A moderately diluted enzyme was added to initiate the reaction. Then 200 µL of 1 M Na_2_CO_3_ solution was added to terminate the reaction. Reaction tubes added with heat-inactivated enzyme were used as control. The reaction solution was transferred to a 0.5 mm colorimeter to read the absorbance at 405 nm. One unit of enzyme activity was defined as the activity of enzyme needed to hydrolyze *p*NP-β-Glc to produce 1 μ mol *p*NP per minute under 90 °C and pH 6.0 condition.

The enzyme activities of β-glucosidase TsBgl1 on disaccharide substrates such as cellobiose, sophorose, gentiobiose and laminaribiose were determined by the glucose oxidase-peroxidase (GOD-POD) method. The glucose content in the reaction solution was determined by a GOD kit. 1 mL’s GOD glucose oxidase reagent was added with a 70 μL properly diluted sample, and reacted at room temperature for 10 min. Then, 200 μL reaction solution was absorbed into the enzyme plate and put into the spectrophotometer to determine its absorbance at wavelength 505 nm. The glucose concentration in the sample was calculated according to the standard curve. One unit of enzyme activity was defined as the activity of enzyme required to hydrolyze the disaccharide substrate to release 2 μmol of glucose per minute under 90 °C and pH 6.0 condition. Each assay was performed in triparallel experiments.

### 2.4. Enzymatic Properties of Recombinant TsBgl1

Using *p*NP-β-Glc as substrate, the enzyme activities were measured at 30–100 °C to determine the optimum reaction temperature. Then under optimum temperature, the enzyme activities in pH 3.0–10.0 buffers were measured to determine the optimal reaction pH of the enzyme. In order to analyze the thermal stability of recombinant TsBgl1, the residual enzyme activities after incubation at different temperatures for a certain period of time were determined under the optimum pH or temperature condition. In order to analyze the stability of recombinant enzyme TsBgl1 in different pH environments, the enzyme solution was placed in buffers of different pH values and stored at 4 °C for seven days, and then the residual enzyme activities were measured under the optimal conditions.

The specific activities of recombinant TsBgl1 against different substrates were determined by using *p*NP-β-Glc, cellobiose, sophorose, gentiobiose and laminaribiose as substrates, respectively. The reactions were carried out with different concentrations of the above-mentioned substrates to determine the enzymatic reaction rates of different substrates, and the kinetic parameters of the enzymatic reaction. The Michaelis constant and catalytic constant were calculated by data fitting using GraphPad Prism version 6.0 software (GraphPad Software, San Diego, CA, USA). In order to determine the glucose tolerance of recombinant TsBgl1, different concentrations of glucose were added at pH 6.0 and 90 °C to make the concentration of glucose in the reaction system between 100 mM–3 M, and the hydrolase activities were determined using *p*NP-β-Glc as substrate.

### 2.5. Optimization of Transglycosylation Reaction Conditions

The enzymatic preparation of GnOS was performed in a 10 mL reaction system consisting of phosphate-citric acid buffer, recombinant TsBgl1 and glucose substrate. The reaction centrifuge tubes were put into a water bath shaker at a certain temperature and sampled at a certain time interval. To explore the effect of reaction temperature on the yield of GnOS, 500 U·g glucose^−1^ of recombinant TsBgl1 was added to react in pH 6.0 citric acid-phosphate buffer at 60–90 °C using 800 g·L^−1^ glucose as the substrate. Similarly, the reactions were carried out under pH 4.0–6.0 to explore the effect of reaction pH, and carried out with different enzyme addition to explore the effect of enzyme amount, respectively. Finally, 300–1000 g·L^−1^ glucose were used as substrates to explore the effect of substrate concentration on the yield of GnOS.

### 2.6. Products Analysis

The reaction samples were boiled for inactivation, centrifugated to get the supernatant, and then diluted with ultrapure water. The composition of these samples was analyzed by high-performance liquid chromatography (HPLC). Agilent 1200 liquid chromatograph differential detector was used. The chromatographic column was HYPERSILAPS2 column (250 × 4.6 mm, 5 μm). The column temperature was set to 35 °C, and the flow rate was 0.8 mL·min^−1^. The mobile phase was 78% acetonitrile/aqueous solution.

## 3. Results and Discussion

### 3.1. Expression, Purification and Characterization of β-Glucosidase TsBgl1

**Figure 1:**
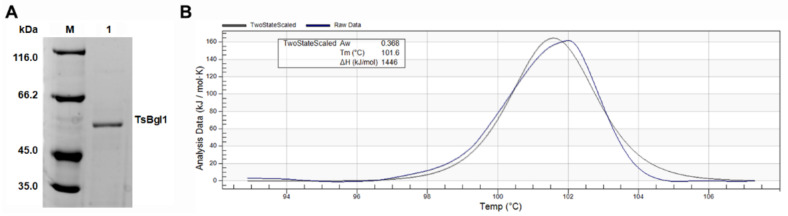
SDS-PAGE (**A**) and DSC (**B**) analysis of purified TsBgl1. M, standard protein marker. Lane 1, purified TsBgl1. Aw, water activity. Tm, melting temperature. ΔH, enthalpy change.

β-glucosidase TsBgl1 was successfully expressed in the expression host *B. subtilis* WS11. The recombinant enzyme solution was purified by ammonium sulfate fractional precipitation and Ni^2+^ affinity chromatography (as shown in Table S2). An obvious single band with a theoretical molecular weight of TsBgl1 was shown in the SDS-PAGE blot (Figure 1A). The results of differential scanning calorimeter (DSC) analysis showed that the melting temperature (T_m_) of recombinant TsBgl1 was 101.5 °C (Figure 1B), which indicated its high thermostability.

**Figure 2:**
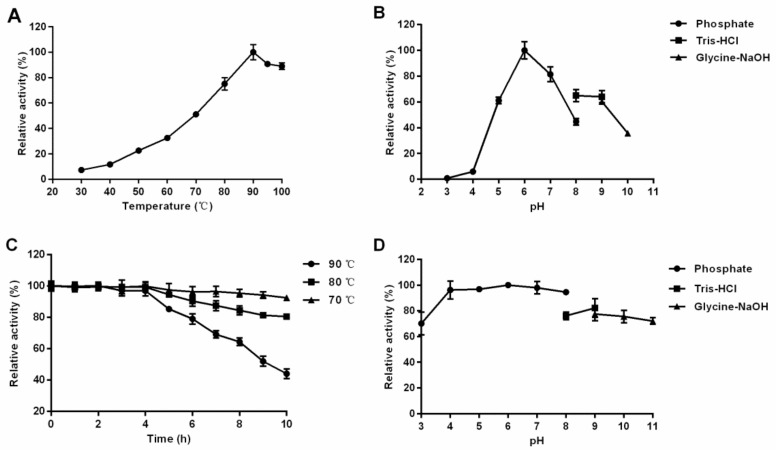
Enzymatic properties of recombinant TsBgl1. (**A**) effect of temperature on enzyme activity. (**B**) effect of pH on enzyme activity. (**C**) thermostability. (**D**) pH stability. The data of pH stability was shown as the residual enzyme activities measured under optimal conditions of enzymes incubated in buffers of different pH values at 4 °C for seven days. The activity assays were performed using 4 mM *p*NP-β-Glc as substrate.

The optimum temperature of recombinant β-glucosidase TsBgl1 was 90 °C, and more than 70% of the enzyme activity could be retained when the reaction temperature was between 70 °C and 100 °C (Figure 2A). When the temperature was lower than 50 °C, the relative activity decreased to less than 20%, indicating that TsBgl1 was a typical thermophilic enzyme. In terms of thermostability, the recombinant TsBgl1 almost did not lose its activity within 4 h after incubation at temperatures of 70–90 °C, and retained more than 60% of the maximum activity after incubation for 8 h (Figure 2C). Moreover, the half-life of recombinant TsBgl1 was approximately 5 h at 90 °C, suggesting its high heat resistance as well. When the reaction temperature was set as 90 °C, TsBgl1 showed the highest enzyme activity in the 50 mM phosphate citrate buffer of pH 6.0, and maintained more than 80% of the maximum activity in the range of pH 5.0–7.0 (Figure 2B). When the pH value was lower than 5.0, the TsBgl1 enzyme activity decreased rapidly. In addition, after the recombinant enzyme TsBgl1 was incubated in different buffers of pH 4.0–9.0 for seven days, all the residual enzyme activities were more than 80% (Figure 2D), indicating that TsBgl1 had good pH stability. The thermophilic and stable properties of TsBgl1 are significant advantages in industrial applications, where long periods of high-temperature reactions are required.

### 3.2. Substrate Specificities and Kinetic Parameters

**Figure 3:**
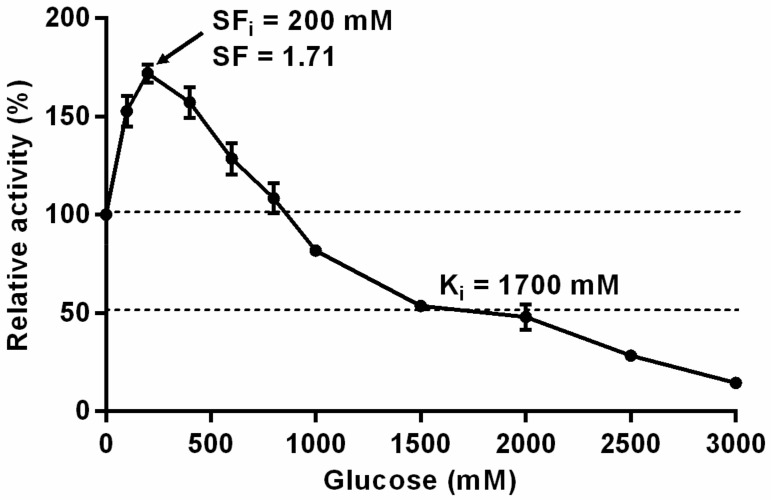
Glucose inhibition curve of recombinant TsBgl1. The activity assays were performed under the optimum condition of 90 °C and pH 6.0 using 4 mM *p*NP-β-Glc as substrate in the presence of different concentrations of glucose.

As shown in Table 1, among all determined substrates, TsBgl1 showed the highest specific activity and catalytic efficiency on *p*NP-β-Glc. Moreover, TsBgl1 had significantly higher specific activity and catalytic efficiency on laminaribiose than other disaccharides. In terms of substrate affinity, the *K*_m_ value of TsBgl1 on gentiobiose and sophorose were the highest, indicating that higher substrate concentration was needed for the effective hydrolysis of these two disaccharide substrates. It was well known that the activity of many β-glucosidase could be competitively inhibited by glucose. The decrease of the catalytic activity of β-glucosidase caused by this inhibition may greatly limit the application of β-glucosidase. The results of glucose tolerance analysis of recombinant TsBgl1 showed that, when the glucose concentration was in the range of 0–900 mM, the activity of TsBgl1 was higher than that without glucose (Figure 3). The maximum activity was observed at a glucose concentration of 200 mM and the stimulating factor (SF) was 1.71. When the concentration of glucose was higher than 900 mM, the activity began to be inhibited by glucose and decreased gradually. The inhibition constant (*K*_i_), which represents the concentration of inhibitor needed to reduce the enzyme activity to half of the maximum activity, was 1.72 M. Several thermostable and glucose tolerant β-glucosidases have been found in recent years. A novel GH1 β-glucosidase Bgl_M_ identified from a hot-spring metagenome showed good thermal stability and remained at 80% relative activity at 1 M glucose concentration [25]. Moreover, the GH3 β-glucosidase produced by *Malbranchea pulchella* (MpBgl3) was reported to be not inhibited by 1 M glucose [26]. Recently, a research group of Amit Ghosh has proved that the presence of glucose binding sites and the accompanying conformational changes was responsible for the uncompetitive inhibition and high tolerance of GH1 β-glucosidase (H0HC94) from *Agrobacterium tumefaciens* 5A [27,28].

### 3.3. Optimization of Reaction Conditions for the Synthesis of GnOS by TsBgl1

**Figure 4:**
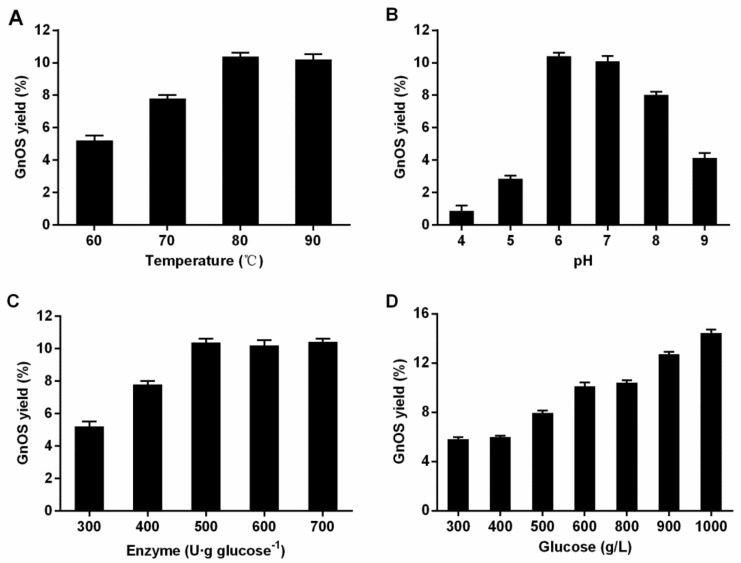
Effects of reaction parameters and substrate concentration on GnOS yield. (**A**) effect of reaction temperature on GnOS yield. (**B**) effect of reaction pH on GnOS yield. (**C**) effect of enzyme amount on GnOS yield. (**D**) effect of substrate concentration on GnOS yield.

In this study, the optimal conditions for the preparation of GnOS catalyzed by TsBgl1 were studied in the aspects of temperature, pH, amount of enzyme and substrate concentration (Figure 4). As the solubility of glucose in water increases with the increase of reaction temperature, high temperature can ensure the complete dissolution of the high concentration substrate in the system and reduce the solution viscosity. So, temperature is an important parameter affecting the preparation of GnOS catalyzed by TsBgl1. The results showed that the yield of GnOS reached the highest value at 80 °C, and the yield changed little with a further increase of temperature, which may be due to the relative poor thermostability of TsBgl1 at 90 °C and the decrease of enzyme activity in the later stage of the reaction. Then the reaction was carried out at 80 °C to explore the effect of different pH values on the yield of GnOS. When pH was below 6.0, the yield increased with the increase of pH, reaching the highest value under pH 6.0, and then decreased with the increase of pH. Furthermore, The balance between the activity of transglycosylation and hydrolysis of the enzyme was another key factor to determine the yield of GnOS. Too little enzyme will lead to low transglycosylation reaction activity and a low GnOS synthesis rate, while too much enzyme will lead to enhanced hydrolysis and secondary hydrolysis of the product which might terminate the GnOS accumulation. The experimental results showed that GnOS reached the highest yield when the amount of TsBgl1 was 500 U·g glucose^−1^. Moreover, the yield of GnOS remained basically unchanged when the amount of enzyme increased, indicating that the activity of transglycosylation and hydrolysis reached a balance at this dosage. Because a high concentration of glucose can competitively inhibit the hydrolytic activity of the enzyme and significantly reduce the water activity, the effect of substrate concentration on the yield of GnOS is also very important. In this study, the reaction was carried out with 300–1000 g·L^−1^ glucose as substrates at 80 °C and pH 6.0. The results showed that the yield of GnOS increased with the increase of glucose concentration. When the substrate concentration is 800 g·L^−1^ glucose, the yield of GnOS was 10.41%. Moreover, when the substrate concentration was increased to 1000 g·L^−1^, the yield of GnOS reached the highest value of 14.43%.

### 3.4. Reaction Time Course and Product Composition Analysis of TsBgl1

**Figure 5:**
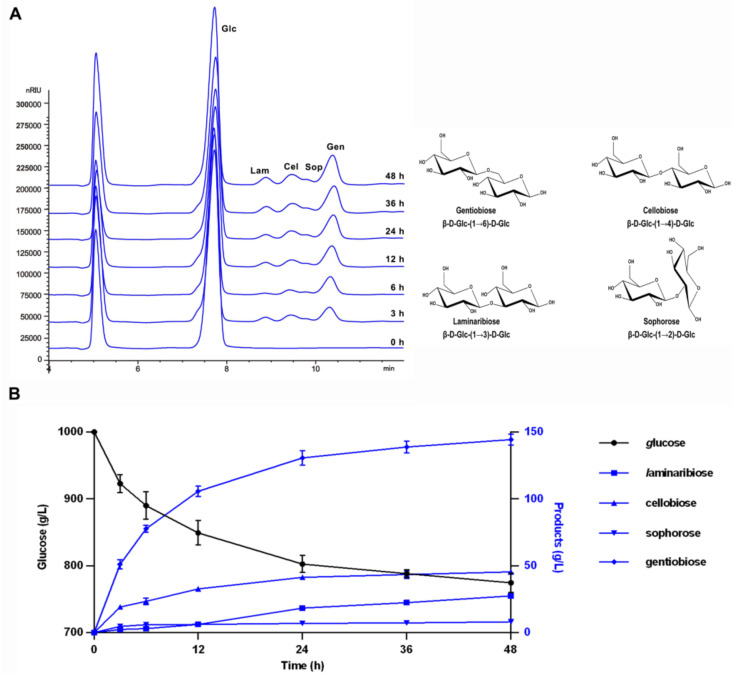
Transglycosylation product analysis of recombinant TsBgl1. (**A**) HPLC chromatograms of transglycosylation products and formulas of the disaccharides synthetized by TsBgl1. Lam, laminaribiose (Glc-β-1,3-Glc). Cel, cellobiose (Glc-β-1,4-Glc). Sop, sophorose (Glc-β-1,2-Glc). Gen, gentiobiose (Glc-β-1,6-Glc). The formulas of the disaccharides were shown in the boat conformation, and the position of the linked hydroxyl group from the non-reducing end to the reducing end were annotated by arrow. (**B**) Time course of TsBgl1 catalyzed transglycosylation. The reaction was performed by adding 500 U·g glucose^−1^ TsBgl1 and using 1000 g·L^−1^ glucose as substrates at 80 °C and pH 6.0.

Using 1000 g·L^−1^ glucose as substrate, the enzymatic transglycosylation reaction was carried out by adding 500 U·g glucose^−1^ TsBgl1 at 80 °C and pH 6.0. During the progress of the reaction, the glucose concentration decreased and the transglycosylation products accumulated gradually. Four kinds of disaccharides were detected in the product (Figure 5A), among which the content of gentiobiose was the highest and the content of sophorose was the lowest. It suggested that TsBgl1 was able to synthesize disaccharides with four kinds of glucosidic bonds at the same time. When the reaction continued to 48 h, the concentration of transglycosylation products reached the highest yield (Figure 5B). Early researchers have been reported to use the reverse hydrolysis or condensation reaction catalyzed by β-glucosidase to prepare GnOS from high concentration glucose syrup. The yield of GnOS was roughly distributed between 5–15% at 200–900 g·L^−1^ glucose substrate concentration (Table 2). Da Silva et al. [29] synthesized 128 g·L^−1^ gentiobiose by a β-glucosidase from *Prunus dulcis* (β-Pd) using 900 g·L^−1^ glucose as substrate. Wang et al. isolated a β-glucosidase with high transglycosylation activity from *Trichoderma viride* and yielded 130 g·L^−1^ GnOS from 800 g·L^−1^ glucose [30], which was the highest yield ever published. In this study, the highest yield of GnOS synthesized by recombinant TsBgl1 was 143.3 g/L, which was higher than the highest yield reported previously. Moreover, TsBgl1 showed obvious advantages in industrial-scale continuous production for its excellent thermophilic and thermostable properties. Therefore, β-glucosidase TsBgl1 derived from *Thermotoga* sp. KOL6 was considered to be a dominant candidate enzyme for the enzymatic synthesis of GnOS and had a good application prospect.

## 4. Conclusions

With the improvement of modern human living standards, energy excess caused by a high-calorie diet is causing more and more common sub-health and chronic diseases. A low-calorie diet has become a new public demand, which promotes the functional oligosaccharides containing anti-digestive glycosidic bonds to become the research focus of new food components. In this work, the enzymatic properties of recombinantβ-glucosidase TsBgl1 from *Thermotoga* sp. KOL6 were characterized, and its performance in enzymatic preparation of GnOS was evaluated. TsBgl1 exhibited excellent thermophilic and thermostable properties by possessing a T_m_ of 101.5 °C and reacting at 80–90 °C efficiently, which was beneficial to continuous production at high temperatures. By optimizing the reaction conditions and substrate concentration, it was found that when 1000 g·L^−1^ glucose was used as a substrate, the highest yield of GnOS synthesized by TsBgl1 could reach 144.3 g·L^−1^, which was higher than the highest yield ever reported. It can be seen that TaAglA showed considerable potential application value. This study was expected to provide a reference for studying the transglycosylation of GH1 β-glucosidases.

## Figures and Tables

**Table 1:** Specific activities and kinetic parameters of recombinant TsBgl1 on different substrates.

Substrate	Specific Activity (U/mg)	*K*_m_ (mM) ^a^	*k*_cat_ (s^−1^) ^b^	*k*_cat_/*K*_m_ (mM^−1^·s^−1^)
*p*NP-β-Glc	181.3	0.24	123.7	509.1
Cellobiose (Glc-β-1,4-Glc)	85.5	2.13	246.2	115.6
Sophorose (Glc-β-1,2-Glc)	91.2	3.95	218.2	55.3
Gentiobiose (Glc-β-1,6-Glc)	87.8	3.68	240.0	65.2
Laminaribiose (Glc-β-1,3-Glc)	140.8	2.17	295.0	135.7

^a^ *K*_m_ value was the Michaelis constant. ^b^ *k*_cat_ value was the turnover number.

**Table 2:** Current status of enzymatic preparation of GnOS.

Source Species	Substrate	Yield (g·L^−1^) ^a^	Reference
*Thermotoga* sp. KOL6	1000 g·L^−1^ glucose	144.3	This work
*Prunus dulcis*	900 g·L^−1^ glucose	128	Da Silva et al. [29]
*Penicillium multicolor*	500 g·L^−1^ gentiotriose	157	Fujimoto et al. [1]
*Fusarium solani*	30 g·L^−1^ cellobiose	16.8	Boudabbous et al. [31]
*Aspergillus niger*	17.1 g·L^−1^ cellobiose	6.35	Seidle et al. [32]
*Fusarium oxysporum*	200 g·L^−1^ cellobiose	28.2	Christakopoulos et al. [33]
*Trichoderma viride*	800 g·L^−1^ glucose	130	Wang et al. [30]

^a^ Some of the presented data were obtained through calculating conversion.

## Data Availability

Data is contained within the article or supplementary material.

## Supplementary Materials

The following are available online at https://www.mdpi.com/article/10.3390/foods11030357/s1, Table S1: Primers used in this study, Table S2: Purification of recombinant TsBgl1, Figure S1: Fitting curves of enzyme kinetics of TsBgl on different substrates.

## Abbreviations

GnOS	Gentiooligosaccharides
GH	Glycoside hydrolase
*p*NP-β-Glc	4-nitrophenyl-β-D-glucopyranoside
GOD-POD	Glucose oxidase-peroxidase
DSC	Differential scanning calorimeter
SF	Stimulating factor
*K*_i_	Inhibition constant
