# Supplementary material for: Enzymatic Preparation of Gentiooligosaccharides by a Thermophilic and Thermostable β-Glucosidase at a High Substrate Concentration
Source: Foods. 2022 Jan 26;11(3):357. doi: 10.3390/foods11030357 (PMC8834124; doi:10.3390/foods11030357)
Supplement: Supplementary file 1 [file foods-11-00357-s001.zip › foods-1517310-supplementary.pdf]

Supplementary Materials

Table S1. Primers used in this study.

Primers	Primer sequence (5′→3′)	
	Forward	Reverse
TsBgl1	TAAGGAGTGTCAAGAATGAGCATGAAAAAGTTTCCGGAAG	TTTATTACCAAGCTTTTAATCTTCCAGGCCGTTATTTTTAATAAC
pBSMμL3	AAGCTTGGTAATAAAAAAACACCTC	CATTCTTGACACTCCTTATTTG

Table S2. Purification of recombinant TsBgl1.

Prufication procedure	Total protein (mg)	Total activity (U)	Specific activity (U/mg) [a]	Yield (%)	Purification fold
Crude enzyme	421.3	7541.3	17.9	100.0	1.0
$(NH_4)_2SO_4$	76.6	5453.9	71.2	72.3	4.0
Ni^{2+} affinity	15.6	2828.3	181.3	37.5	10.1

[a] The specific activity was determined using *p*NP-β-G as the substrate.

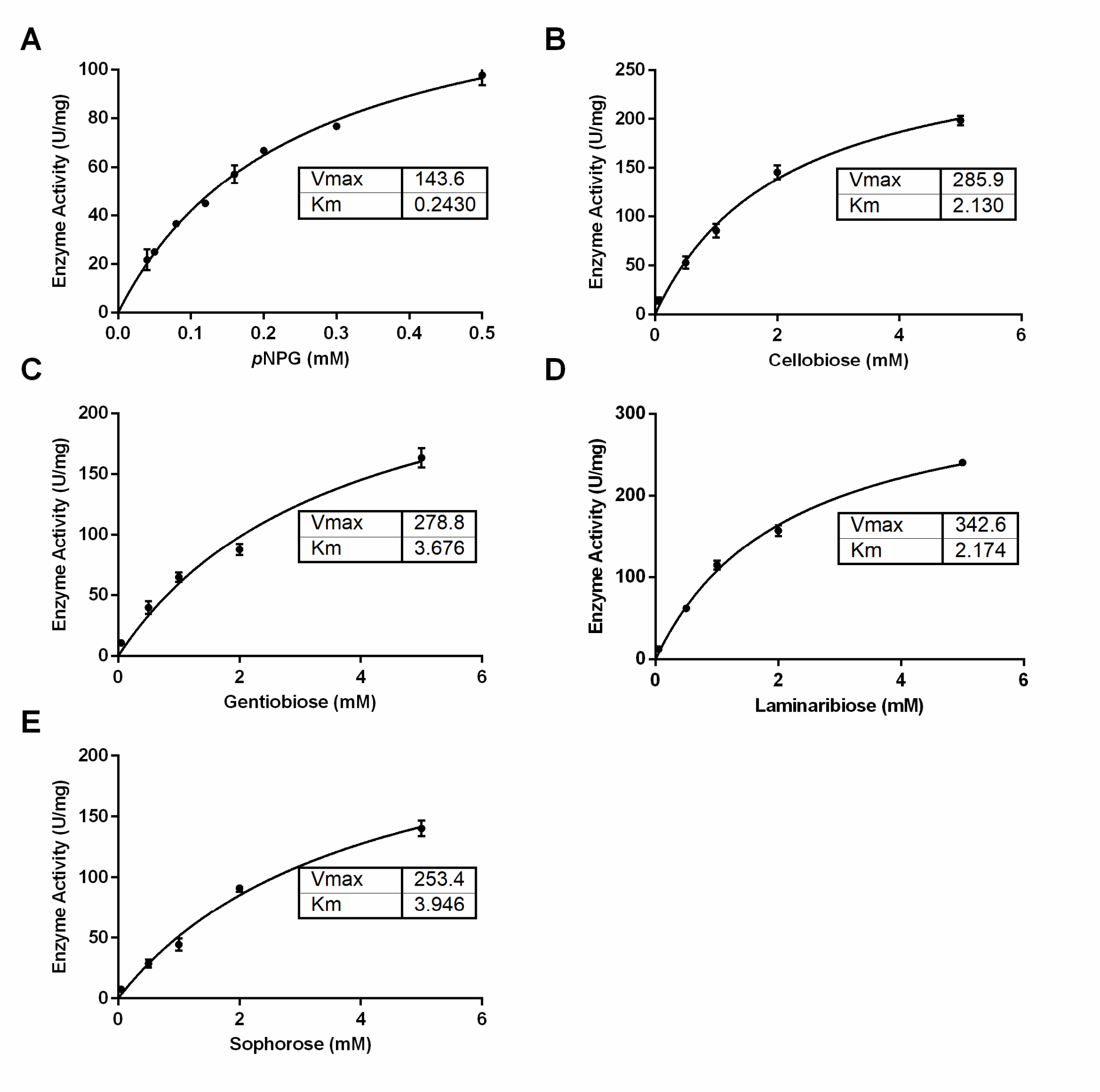

Figure S1. Fitting curves of enzyme kinetics of TsBgl on different substrates. A, *p*-nitrophenol. B, cellobiose. C, gentiobiose. D Laminaribiose. E, sophorose. Vmax represents the theoretical value of the maximum reaction rate that can be achieved with increasing substrate concentration. *K*m represents the Michaelis constant.
